# Predictive Feature Generation and Selection Using Process Data From PISA Interactive Problem-Solving Items: An Application of Random Forests

**DOI:** 10.3389/fpsyg.2019.02461

**Published:** 2019-11-21

**Authors:** Zhuangzhuang Han, Qiwei He, Matthias von Davier

**Affiliations:** ^1^Teachers College, Columbia University, New York, NY, United States; ^2^Educational Testing Service, Princeton, NJ, United States; ^3^National Board of Medical Examiners, Philadelphia, PA, United States

**Keywords:** process data, interactive items, feature generation, feature selection, random forests, problem-solving, PISA

## Abstract

The Programme for International Student Assessment (PISA) introduced the measurement of problem-solving skills in the 2012 cycle. The items in this new domain employ scenario-based environments in terms of students interacting with computers. Process data collected from log files are a record of students’ interactions with the testing platform. This study suggests a two-stage approach for generating features from process data and selecting the features that predict students’ responses using a released problem-solving item—the Climate Control Task. The primary objectives of the study are (1) introducing an approach for generating features from the process data and using them to predict the response to this item, and (2) finding out which features have the most predictive value. To achieve these goals, a tree-based ensemble method, the random forest algorithm, is used to explore the association between response data and predictive features. Also, features can be ranked by importance in terms of predictive performance. This study can be considered as providing an alternative way to analyze process data having a pedagogical purpose.

## Introduction

Computer-based assessments (CBAs) are used for more than increasing construct validity (e.g., Sireci and Zenisky, 2006) and improving test design (e.g., van der Linden, 2005) through inclusion of adaptive features. They also provide new insights into behavioral processes related to task completion that cannot be easily observed using paper-based instruments (Goldhammer et al., 2013). In CBAs, a variety of timing and process data accompany test performance. This means that much more data from the response process of an answer is available in addition to correctness or incorrectness.

Along with assessing the core domains of Math, Reading, and Science, the Programme for International Student Assessment (PISA) introduced a problem-solving domain in the 2012 cycle, with fundamental technical support from computer delivery. It enabled interactive problems – problems in which exploration is required to uncover undisclosed information (Ramalingam et al., 2014)—to be included in a large-scale international assessment for the first time (Organisation for Economic Co-operation and Development [OECD], 2014b). Dynamic records of actions generated during the item-response process form a distinct sequence representing participants’ input and the internal state of the assessment platform. Analyzing these sequences can facilitate understanding of how individuals plan, evaluate, and select operations to achieve the problem-solving goal (e.g., Goldhammer et al., 2014; Hao et al., 2015; He and von Davier, 2016; Liao et al., 2019).

The problem-solving items in this new domain were typically designed as interactive tasks. The contents of these items cover a broad scope, from choosing an optimal geographic path between departure and destination points to purchasing metro tickets via a vending machine. Both the students’ responses and the whole process of how students solved the problem in a sequence were captured in log files, such as clicking buttons, drawing lines, dragging on a scale, performing keystrokes to respond to open-ended items, and so on. The data contained in log files, referred to as *process data* in the present study, provide information beyond response data (i.e., whether the final response was correct or not).

While process data are expected to provide a broader range of information, the complex embedded structure demands an extension of existing analysis methods. These demands entail efforts to apply techniques used in other disciplines such as data mining, machine learning, natural language processing (NLP), social networking, and sequence data mining. These techniques serve two purposes: (1) creating predictive features/variables^1^ associated with an outcome variable (i.e., feature generation) and (2) determining which features are the most predictive (i.e., feature selection).

The present study analyzed process data from a released PISA 2012 item (Organisation for Economic Co-operation and Development [OECD], 2014a)—Climate Control Task – that is intended to measure problem-solving skills of participants in science. The purpose of this study was twofold: first, to use process data obtained in a simulation-based environment to generate predictive features; and second, to identify the most important predictive features associated with success or failure on the task. The present study employed one of the tree-based ensemble methods – random forests – to select the most predictive features when considering students as the target of inferences.

The remainder of this paper is organized as follows. First, a brief overview of the methods is provided for generating features from process data and selecting important classifiers. The random forest algorithm is introduced and its potential use in analyzing process data is discussed. In the subsequent section, an integrated approach for generating features from process data and selecting features by the algorithm is introduced using a case study from the PISA 2012 problem-solving item. Results obtained from the introduced approach and their interpretations are then presented. Lastly, thoughts on the limitations and implications of the suggested approach are given.

## Overview of Feature Generation and Selection Using Process Data

### Generating Features Using Process Data

The principle of predictive feature generation is to maximize information exploration generated solely from timing and process data. This information may be indicative of respondents’ problem-solving processes, which are associated with the problem-solving skills targeted in the assessment. As summarized in He et al. (2018), the features collected in log files can be roughly categorized into three groups: (1) behavioral indicators that represent respondents’ problem-solving strategies and interactions with the computer, (2) sequences of actions and mini-actions that are directly extracted from test takers’ process data, and (3) timing data such as total time on task, duration of respondent actions in the simulation environment, and time until first actions are taken by the respondent when solving a digital task.

#### Behavioral Indicators

Behavioral indicators are typically recorded at a higher, aggregated level. Although human-computer interactions are often accomplished through simple gestures or movements, in most cases, they are not automated actions but involve case-based reasoning and self-regulatory processes (Shapiro and Niederhauser, 2004; Azevedo, 2005; Lazonder and Rouet, 2008; Zimmerman, 2008; Brand-Gruwel et al., 2009; Bouchet et al., 2013; Winne and Baker, 2013). Therefore, to perform well on computer-based problem-solving tasks, one needs to have essential skills in using information and communication technology tools and higher-level skills in problem solving. Respondents have to decode and understand menu names or graphical icons in order to follow the appropriate chain of actions to reach a goal. Meanwhile, problem-solving tasks also require higher-order thinking, finding new solutions, and interacting with a dynamic environment (Mayer, 1994; Klieme, 2004; Mislevy et al., 2012; Goldhammer et al., 2014).

A typical example is the strategy indicator “vary one thing at a time (VOTAT)” studied in Greiff et al. (2015). This study showed that VOTAT was highly correlated with student performance. Note that solving complex, interactive tasks requires developing a plan consisting of a set of properly arranged subgoals and performing corresponding actions to attain the final goal. This differs from solving logical or mathematical problems, where complexity is determined by reasoning requirements but not primarily by the information that needs to be accessed and used (Goldhammer et al., 2013). In this sense, one could argue that the indicators of user actions should in some systematic way map onto the subgoals a user develops and applies to achieve a successful completion of the learning or assessment task.

Another example of a strategy indicator was derived from the problem-solving path and pace of examinees as studied in Lee and Haberman (2016). In this study, it was found that test takers adopted different strategies in solving reading tasks in an international language assessment and that these strategies were highly related to respondents’ country, language, and cultural background. For example, the typical strategy of test takers from two Asian countries was to skip the passage and view the questions first. Based on what the item’s instructions requested, those test takers went back to read the passage and locate the information needed. Conversely, participants from two European countries were found to follow what was intended, that is, read the stimuli passage first and then answer the questions. These two strategies did not have a significant relationship to performance of test takers, although substantial performance differences and completion rates were found in the low-performing group.

#### Sequences of Actions From Process Data

The importance of sequence data in education has been recognized for decades. Agrawal and Srikant (1995) said “the primary task, as applied in a variety of domains including education, is to discover patterns that are found in many of the sequences in a dataset.” Actions or mini-sequences that can be represented as *n*-grams (He and von Davier, 2015, 2016) are typical indicators to describe respondents’ behavioral patterns. For instance, actions related to “cancel” (e.g., clicking on a cancel button in order to go back and change or check entries again) are sequence indicators, which are associated with test takers’ cognitive processes and may indicate hesitation or uncertainty about next steps. Mini-sequences can not only show the actions adjacent to each other, but also the strategy link between the actions. For example, in He and von Davier (2016), strategy changes between the searching and sorting functions were successfully identified through analysis of bigrams and trigrams. More details on the use of *n*-grams for analyzing action sequences are given in the see section “Materials and Methods”.

Some researchers have employed sequential pattern mining to inform student models for customizing learning to individual students (e.g., Corbett and Anderson, 1995; Amershi and Conati, 2009). Other researchers have employed sequential pattern mining to better understand groups’ learning behaviors in designed conditions (e.g., Baker and Yacef, 2009; Zhou et al., 2010; Martinez et al., 2011; Anderson et al., 2013). As Kinnebrew et al. (2013) summarized, “identifying sequential patterns in learning activity data can be useful for discovering, understanding, and, ultimately, scaffolding student learning behaviors.” Ideally, these patterns provide a basis for generating models and insights about how students learn, solve problems, and interact with the environment. Algorithms for mining sequential patterns generally associate some measures of frequency to rank identified patterns. The frequency of a pattern along the problem-solving process timeline can provide additional information for interpretation. Further, the observed variability across action-sequence patterns may play an important role in identifying behavioral patterns that occur only during a certain span of time or become more or less frequent than ones occurring frequently but uniformly over time, thus allowing us to explore what conditions lead to such changes (Kinnebrew et al., 2013).

Sequential pattern mining can be conducted via various approaches. For instance, Biswas et al. (2010) used hidden Markov models (HMMs; Rabiner, 1989; Fink, 2008) as a direct probabilistic representation of the internal states and strategies. This methodology facilitated identification, interpretation, and comparison of student learning behaviors at an aggregate level. As with students’ mental processes, the states of an HMM are hidden, meaning they cannot be directly observed but produce observable output (e.g., actions in a learning environment).

As Fink (2008) pointed out, the development and spread in use of sequential models was closely related to the statistical modeling of texts as well as the restriction of possible sequences of word hypotheses in automatic speech recognition. Motivated by the methodologies and applications in NLP and text mining (e.g., He et al., 2012; Sukkarieh et al., 2012), a number of methods from these fields can be borrowed for application in process data analysis. For instance, the longest common subsequence introduced by Sukkarieh et al. (2012) to educational measurement for scoring computer-based Program for the International Assessment of Adult Competencies items was used in He et al. (2019) to extract the most likely strategy by respondent in each item by calculating the distance between individual sequences and reference ones. This approach allowed comparisons of respondents’ behavior across multiple items in an assessment.

#### Features Generated From Timing Data

In addition to sequential data on actions taken by respondents during the problem-solving process, CBAs provide rich data on response latency or timing data. Each action log entry is associated not only with data on what a respondent did, but also when the action took place. These timestamps can be aggregated to an overall time measure for the survey, which is specific to the task, or measures that are specific to certain types of interactions such as keystrokes, navigation behavior, or time taken for reading instructions. Timing data at this level of resolution has led to renewed interest in how latency can be used in modeling response processes (e.g., DeMars, 2007; van der Linden et al., 2010; Weeks et al., 2016). In addition, timing data information is expected to be valuable in conjunction with the types of actions observed in the sequence data and to help us derive features that allow predicting cognitive outcomes such as test performance as well as background variables (Liao et al., 2019).

### Predictive Feature Selection

Feature selection models play an essential role in identifying predictive indicators that can distinguish different groups, such as the correct and incorrect groups at the item level in problem-solving processes. A variety of models that have been developed in “big data” fields that relate to information retrieval, NLP, and data mining are also applicable to process data analysis. Here, we discuss some commonly used feature selection models that are popularly used in similar settings, ultimately focusing on one tree-based ensemble method – the random forest method – which will be further applied in this study.

As reviewed by Forman (2003) as well as Guyon and Elisseeff (2003), the feature selection approaches are essentially divided into wrappers, filters, and embedded methods. *Wrappers* utilize the learning machine of interest as a black box to score subsets of variables according to their predictive power. *Filters* select subsets of variables as a preprocessing step, independent of the chosen predictor. *Embedded* methods perform variable selection in the process of training and are usually specific to given learning machines. We introduced these three methods in details in the following subsections. In the embedded methods, the random forests method that has been used in this study is highlighted.

#### Wrapper Methods

These methods, popularized by Kohavi and John (1997), offer a simple and powerful way to address the problem of variable selection, regardless of the chosen machine learning approach. In their most general formulation, they consist of using the prediction performance of a given approach to assess the relative usefulness of subsets of variables. The wrapper methods that are most used in sequential forward selection or genetic search perform an exhaustive search over the space of all possible subsets of features, “repeatedly calling the induction algorithm as a subroutine to evaluate various subsets of features” (Guyon and Elisseeff, 2003). These methods are more practical for low-dimensional data but often are not for more complex large-scale problems due to intractable computations (Forman, 2003).

#### Filter Methods

These methods apply an intuitive approach in that the associations of each predictor variable with the response variable are individually evaluated, and those most associated with it are selected. For nominal response variables (the case considered in this study), measures of dispersion (also referred to as concentration or impurity depending on the context) such as Gini’s impurity index and Shannon (1948)’s entropy are employed as the building blocks for measures of association between response variables and features (Haberman, 1982; Gilula and Haberman, 1995). In cases where response and features are both categorical, Goodman and Kruskal (1954) measure the association using the proportion of reduction of concentration if a predictor variable is involved. Other examples of measures of association can be found in, Theil (1970), Light and Margolin (1971), and Efron (1978).

Practices in area such as NLP implement an even more simplified approach by comparing the value of test statistics of association such as the chi-square statistic for the nominal response and categorical independent variable (Nigam et al., 2000; Oakes et al., 2001; He et al., 2012, 2014). Though some have raised concerns that this approach lacks statistical significance and soundness, its practical effectiveness in ordering the importance of categorical features makes it broadly accepted by certain audiences (Manning and Schütze, 1999; Forman, 2003). Applications can be founded in the recent literature about feature selection in large-scale assessment (He and von Davier, 2015, 2016; Liao et al., 2019).

#### Embedded Methods

These methods incorporate variable selection as part of the model training process. Compared with wrapper methods, they are more efficient and reach a faster solution by avoiding retraining a predictor from scratch for every variable subset investigated (Guyon and Elisseeff, 2003). For instance, the classification and regression tree (CART; Breiman et al., 1984) algorithm can be redesigned to serve this purpose. The random forest algorithm (Breiman, 2001), as an extension of CART that is a random ensemble of multiple trees, belongs to the family of embedded methods and is the method chosen for the current study. The random forest algorithm increasingly adjusts itself by randomly combining a predetermined number of single tree algorithms (shorten as trees in later sections). By aggregating the prediction results obtained from individual trees, the forest reduces prediction variance and improves overall prediction accuracy (Dietterich, 2000).

Some basic ideas about tree algorithms are reviewed here to facilitate understanding of the random forest algorithm. Let *X* = *X*_1_,…,*X*_*p*_ for covariates and *Y* denote the outcome variable. Instead of establishing an analytical form of predicting *Y* from *X*, a decision tree grows by recursively splitting the space of covariates extended by the set *X* in a greedy way such that segments (nodes) created have the least impurity (for classification) or mean squared error (for regression) possible and are thus used to predict*Y*. Binary split – splitting a parent node into two child nodes – is conventionally employed and guided by the splitting rules. For classification, one of the rules is the Gini impurity index (Breiman et al., 1984; Breiman, 2001),

$$I_{G}\,\left(s,t\right)=1-\sum_{k}p_{k}^{2}\,\left(s,t\right),$$

where *t* denotes the current node, *p*_*k*_(*s*,*t*) is the frequency of class *k* in the samples of node *t*, and split *s* represents a certain numeric value or class label of a covariate *X*_*j*_. If *Y* is binary, the above expression will be simplified as $1-p_{0}^{2}\,\left(s,t\right)-p_{1}^{2}\,\left(s,t\right)$. It is intuitive that the index is a measure of dispersion: 1 indicates the utmost dispersion and 0 stands for the most extreme concentration. In other fields such as ecology, the index used to measure diversity is known as the Simpson-Gini Index due to its similarity to the Simpson Index (Peet, 1974). It should be noted that the estimate of *I*_*G*_(*s*,*t*) is biased for small samples if the sample frequencies *f*_*k*_(*s*,*t*)=*n*_*k*_(*s*,*t*)/*n*(*s*,*t*) are directly used. This is because the unbiased estimate of $p_{k}^{2}\,\left(s,t\right)$ is $\frac{n_{k}\,\left(s,t\right)\,\left[1-n_{k}\,\left(s,t\right)\right]}{n\,\left(s,t\right)\,\left[1-n\,\left(s,t\right)\right]}$. A simple modification can be implemented to correct this bias.

The optimal split is determined by seeking the *s* that maximizes

$$\Delta \,I_{G}\,\left(s,t\right)=I_{G}\,\left(s,t\right)-\frac{1}{N_{t}}\,\left[N_{t_{l}}\,I_{G}\,\left(s,t_{l}\right)+N_{t_{r}}\,I_{G}\,\left(s,t_{r}\right)\right]$$

through the given predictors in set *X*. The quantity above indicates the decrease of impurity resulting from splitting the parent node *t* at *s* into the left child node *t*_*l*_ and the right child node *t*_*r*_. Sample sizes (*N*_*t_l_*_ and *N*_*t_r_*_) of child nodes are used to obtain the weighted impurity. For regression, the mean squared error is applied as the splitting rule (Breiman et al., 1984; Breiman, 2001).

Random forests ensemble individual decision trees through the following steps. First, subsets of samples are randomly drawn from the whole sample dataset and individual trees are grown based on each subset of samples. Note that data entries not chosen in each random draw are called “out of bag” data and kept for validating purposes. Second, for each individual decision tree in the random forest algorithm, it grows by recursively splitting a parent node into two or more child nodes with respect to a set of predictor variables as previously discussed. Rather than seeking the “best” cut point through all available predictor variables, the tree of random forests only examines through a set of *m* randomly chosen variables at each split. An individual tree stops to grow when a preset number of leaf nodes (nodes at the end of the tree that have no child nodes) or a threshold in terms of impurity of child nodes is reached. Third, final predicted responses are obtained by aggregating the prediction results over these fitted individual trees constructed using different subsets of covariates.

Even though the stability of an individual tree in terms of prediction is still not quite comparable with a typical logistic regression model fitted using all covariates, Breiman et al. (1984) argued that the variance is reduced because of the aggregation, which further enhances the overall prediction performance. Lin and Jeon (2006) showed that the random forest outperforms other less model-based predictive methods in cases with moderate sample sizes. In addition to the improvement on prediction performance, random forests also have other advantages in practice. As introduced above, only a certain number of covariates are selected to conduct each split when growing a decision tree. Such a feature allows the random forest algorithm to fit with a relatively larger number of predictor variables (especially for categorical variables) on a given sample size compared to other predictive methods such as linear models (e.g., generalized linear models), for which fitting with an extensive number of predictors may create data sparsity and reduce the numerical robustness.

In addition, two built-in variable selection methods of random forests, using two types of variable importance measures (VIMs)—(1) impurity importance and (2) permutation importance – have been successfully applied in fields such as gene expression and genome-wide association studies (Díaz-Uriarte and Alvarez de Andrés, 2006; Goldstein et al., 2011). The current study utilizes the permutation importance to select the most important variables extracted from the process data.

Impurity importance is quantified by accumulating Δ*I*_*G*_(*s*,*t*) for each covariate over nodes of all trees. The accumulation is weighted by the sample sizes of nodes. While the importance measure enjoys all the computational convenience of the random forest algorithm, the splitting mechanism – just by chance – favors variables with many possible split points (e.g., categorical variables with many levels), resulting in a biased variable selection (Breiman et al., 1984; White and Liu, 1994). Much statistical literature further investigated this issue and proposed practical solutions (Kim and Loh, 2001; Hothorn et al., 2006; Strobl et al., 2007; Sandri and Zuccolotto, 2008). For instance, Strobl et al. (2007) reimplemented the random forest method based on Hothorn et al.’s (2006) conditional inference tree-structural algorithms (*ctrees*) to provide unbiased estimation of impurity importance. Instead of altering the algorithm, Sandri and Zuccolotto (2008) proposed a heuristic procedure to directly correct the bias of impurity measure by differentiating the “importance” resulting from characteristics of variables from the importance due to the association with the outcome variable.

As another built-in VIM of the random forest algorithm, the measure of permutation importance is free from this undesirable bias. Although it has been criticized for its computational inconvenience, the simple nature of the permutation importance measure becomes attractive as computation speed increases. The rationale of the permutation importance measure is as follows: First, a predictor variable, say *X*_*j*_, is permutated in terms of the order of samples. Second, together with the other unaltered variables, another random forest algorithm is fit to compare with the algorithm constructed using unaltered samples. Permutation breaks the original association between *X*_*j*_ and *Y*, resulting in a drop of prediction accuracy for the testing data. Lastly, the rank of predictor variables can be established after applying this procedure to each covariate. In the present study, the permutation importance measure, also known as the mean decrease accuracy (Breiman, 2001), was implemented to conduct variable selection.

Tree-based ensemble algorithms also include bagging (Breiman, 1996) and boosting (Freund and Schapire, 1997). Bagging-tree algorithms are similar to random forests but are more straightforward in terms of randomizing the data and growing individual trees. Boosting-tree algorithms grow a sequence of single trees in a way that the latter grown tree fits the variation not explained by the former grown tree. Bayesian additive regression tree (BART; Chipman et al., 2010) is a tree ensemble method established in the Bayesian approach, offering a straightforward means of handling model selection by specifying a prior for the tuning parameter controlling the complexity of trees. Meanwhile, BART considers the uncertainty of parameter estimation with that of model selection. In addition, this method provides a flexible way to address the missing data issue by allowing for directly modeling the missing mechanism.

## Materials and Methods

### Item Description and Data Processing

This study analyzed process data from a computer-based problem-solving item from PISA 2012 – Climate Control Task 1 (item code CP02501). The full-sample data has been made publicly available by the OECD^2^. The dataset for this item includes responses from 30,224 15-year-old in-school students from 42 countries and economies. Sample sizes of countries and economies are shown in Table 1.

**TABLE 1:** Countries and economies with sample sizes.

**Country and economy**	**Sample size**
Australia	1,855
Austria	442
Belgium	726
Bulgaria	988
Canada	1,516
Chile	526
Chinese Taipei	494
Columbia	736
Croatia	962
Czechia	1,526
Denmark	636
Estonia	464
Finland	1,769
France	429
Germany	430
Hong Kong	433
Hungary	424
Ireland	407
Israel	440
Italy	453
Japan	1,005
Korean	449
Macao	519
Malaysia	938
Montenegro	917
Netherland	891
Norway	401
Poland	379
Portugal	486
Russia	504
Serbia	867
Shanghai-China	408
Singapore	469
Slovak	485
Slovenia	667
Spain	885
Sweden	418
Turkey	998
United Arab Emirates	1,023
United States	425
Uruguay	966

**FIGURE 1:**
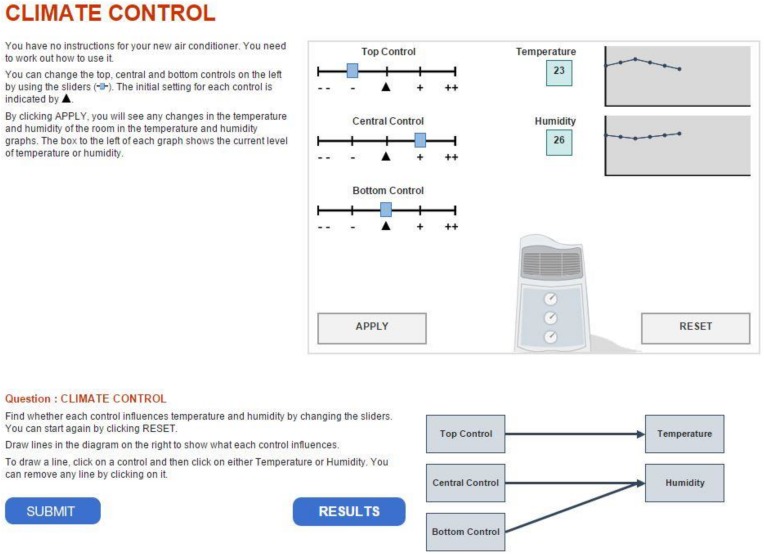
A snapshot of the problem-solving item Climate Control in PISA 2012.

This item (a snapshot of the item is shown in Figure 1) asked test takers to determine which of the three sliders controls temperature and which controls humidity, respectively. To obtain the correct answer, test takers were permitted to manipulate the sliders and monitor changes through the display. The answer to the task was given by drawing lines linking the diagrams to indicate the association between the inputs (sliders) and outputs. The correct solution is shown in Figure 1. The “reset” button undid previous simulations by clearing the display and resetting the sliders to their initial status. No limit was imposed on the number of steps of manipulation or rounds of exploration. Also, no time constraint was imposed on each item; however, the total test time of a test cluster (problem-solving items) was limited to 20 min. Either one or two clusters were randomly given to a participant depending on different assessment designs (Organisation for Economic Co-operation and Development [OECD], 2014b). The order of items in a cluster was fixed, and a former item could not be resumed once the next item had begun. According to different assignments of clusters, the position of Climate Control Task 1 varied across test forms. For this item, the average time spent by students was 125.5 s and the median time was 114.5 s; 95% of examinees spent from 22.2 s to 290.2 s on the item; only 1,149 participants (about 3.8% of the total sample) finished the task in 30 s or less, with a 5.1% rate of correctness. Given these results, later sections of the paper assume that the item is not considered as speeded for this sample in general and position effects, if any, are negligible. However, the analysis of the current study conducted without considering the speeded issue which should be noted as a limitation and further investigated by future research.

Items like Climate Control Task 1 are constructed using the MicroDYN approach (Greiff et al., 2012) that combines the use of the theoretical framework of linear structural equation models to systematically construct tasks (Funke, 2001) with multiple independent tasks to increase reliability. Briefly speaking, a system of causal relations (e.g., the first slider controls temperature) is embedded in a scenario that allows participants to explore input variables and observe the corresponding changes of output variables through a graphical representation. No specific prior domain knowledge is required for this type of task in general. However, examinees need to gain and have command of the knowledge by exploring and experimenting before providing appropriate answers. For such tasks, a strategic knowledge for effective exploration is crucially important (Greiff et al., 2015)—that is, the VOTAT (vary one thing at a time; Tschirgi, 1980) strategy; this term is also known as the control-of-variable strategy (Chen and Klahr, 1999) in developmental psychology.

In PISA 2012, a partial credit assignment – 0 for incorrect, 1 for partially correct, and 2 for correct – was used to score the responses of Climate Control Task 1. Partial credit was given if a student explored the simulation by using the VOTAT strategy efficiently – only varying one control at a time when trying to change the status of each control individually at least once, regardless of actions being in adjacent attempts or in a round before resetting – but failed to correctly represent the association in a diagram.

To show that the VOTAT strategy is strongly related to performance on the item, Greiff et al. (2015) restricted polytomous responses as dichotomous by treating partially correct as incorrect and then investigated the association between the dichotomous responses with the indicator of applying the VOTAT strategy efficiently alongside other covariates. Following the same settings, the present study explored the association between the binary responses and the indicator of the use of the VOTAT strategy together with other covariates created from the process data to find out (1) whether the current partial scoring rubric was still supported by the prediction model (i.e., random forests)—namely, whether the VOTAT variable was still the most associated factor with responses while interacting with other covariates – and (2) whether the rubric was still sufficient compared with the new predictor features extracted from the process data. It should be noted that the restriction of response variable may not be applicable for items that are intended to measure a construct other than the interactive complex problem-solving (Cheng and Holyoak, 1985; Funke, 2001) skills or constructed without using the MicroDYN approach.

Table 2 shows a section of the postprocessed log file—that is, a readable process dataset whose entries are actions listed in chronological order. The even number indicates the actions belong to a certain test taker. The type of action, as well as the corresponding timestamp, was recorded for each action. Among the action types, “apply” represents actions related to manipulation of sliders because, after setting sliders, a test taker needed to hit the “apply” box, as shown in Figure 1, to see the changed value of temperature and humidity displayed. The changed status of sliders was recorded in the columns “top slider,” “central slider,” and “bottom slider.” The value of status ranges from −2 to 2. Similarly, the action type “diagram” represents drawing a line to link diagrams, as shown at the bottom right of Figure 1. The six-digit binary string shown in the table was used to record the association among diagrams that has been established. For example, “100101” indicates that the top slider controls temperature, whereas the central and bottom sliders control humidity.

**TABLE 2:** An example of process data for a test taker solving the climate control item.

**Event**	**Time**	**Event_order**	**Event_ type**	**Top_ slider**	**Central_slider**	**Bottom_slider**	**Temp_value**	**Humid_value**	**Diag_state**
START_ITEM	1288.1	1	start	NULL	NULL	NULL	NULL	NULL	NULL
ACER_EVENT	1291.9	2	reset	0	0	0	25	25	NULL
ACER_EVENT	1338.4	3	apply	1	1	1	27	28	NULL
ACER_EVENT	1346.8	4	apply	1	1	2	29	33	NULL
ACER_EVENT	1350.1	5	apply	1	2	2	31	36	NULL
ACER_EVENT	1354.5	6	apply	2	2	2	35	36	NULL
ACER_EVENT	1361.1	7	apply	2	1	1	36	36	NULL
ACER_EVENT	1361.1	8	reset	0	0	0	25	25	NULL
ACER_EVENT	1375.3	9	diagram	NULL	NULL	NULL	NULL	NULL	000000
ACER_EVENT	1376.2	10	diagram	NULL	NULL	NULL	NULL	NULL	000000
ACER_EVENT	1400.1	11	diagram	NULL	NULL	NULL	NULL	NULL	000000
ACER_EVENT	1402.1	12	diagram	NULL	NULL	NULL	NULL	NULL	000001
ACER_EVENT	1406.8	13	diagram	NULL	NULL	NULL	NULL	NULL	000001
ACER_EVENT	1408.4	14	diagram	NULL	NULL	NULL	NULL	NULL	000101
ACER_EVENT	1410.2	15	diagram	NULL	NULL	NULL	NULL	NULL	000101
ACER_EVENT	1410.6	16	diagram	NULL	NULL	NULL	NULL	NULL	100101
END_ITEM	1416.1	17	end	NULL	NULL	NULL	NULL	NULL	NULL

*“Event” and “event_type” indicate the type of the current action. “Time” and “event_num” show the time point and order of the current action. “Top_slider,” “central_ slider,” and “bottom_ slider” provide information about the status of each control. “Temp_value” and “humid_value” give the simulated results. “diag_state” gives information on the linking among diagrams. Each type of event is abbreviated using a single capital letter: “S” for “start,” “E” for “end,” “R” for “reset,” “A” for “apply,” and “D” for “diagram.” Data source: This table is extracted from “Log-file databases for released PISA 2012 computer-based items data for problem solving” at http://www.oecd.org/pisa/pisaproducts/database-cbapisa2012.htm.*

To facilitate the analysis, observed sequences of actions were collapsed into respective strings. To obtain such a string, each type of action is abbreviated using a single capital letter: “S” for “start,” “E” for “end,” “R” for “reset,” “A” for “apply,” and “D” for “diagram.” It should be noted that consecutive “D” actions were collapsed into a single “D” action because information related to drawing lines to connect the diagrams is not of central interest in the present study. For the sequence of actions shown in Table 2, it can be simplified as “SRAAAAARDE.”

### Feature Generation

In this study, features (predictor variables) extracted from the process data can be summarized in three categories: variables extracted from action sequences using *n*-gram methods, behavior indicators, and time-related variables.

*N*-gram methods decode a sequence of actions into mini-sequences (e.g., a string of *n* letters in length where the letters remain in the same order as the original sequence of actions) and document the number of occurrences of each mini-sequence. Unigrams, analogous to the language sequences in NLP, are defined as “bags of actions,” where each single action in a sequence collection represents a distinct feature. However, unigrams are not informative in term of transitions between actions. Bigrams and trigrams are considered in this study, with action sequences broken down into mini-sequences containing two and three ordered adjacent actions. Note that the *n*-gram method is productive in creating features based on sequence data without loss of much information about the order of sequence. This class of methods has become widely accepted for feature engineering in fields such as NLP and genomic sequence analysis and was recently applied to analyze process data in large-scale assessment (He and von Davier, 2015, 2016). For example, an *n*-gram can break the action string “SRAAAAARDE” into “S(1), A(5), R(2), D(1), E(1)” for unigrams, “SA(1), AR(1), AA(4), RA(1), RD(1), DE(1)” for bigrams, and “SRA(1), RAA(1), AAA(3), AAR(1), ARD(1), RDE(1)” for trigrams, where the numerals in brackets represent the number of occurrences.

Behavior indicators can also be generated from sequences of actions. Changes to input variables (the positions of controls) shed light on participants’ problem-solving strategies and behaviors. As discussed earlier, partial credit was given to students who explored the connection between the inputs and outputs by utilizing the VOTAT (vary one thing at a time) strategy across the three controls at least once. Greiff et al. (2015) treated this scoring rubric as an indicator variable (i.e., VOTAT) and showed that it was highly associated with the probability of answering this item correctly and overall performance on the test.

This study created an ordinal categorical variable with four levels – from 0 to 3 – each number indicating on how many controls a student has used the VOTAT strategy. This ordinal variable was referred to as “VOTAT group” in the analysis. Another variable named “VOTAT num” was created to count the number of times that a student used the VOTAT strategy regardless of which control he or she applied the strategy to. Additionally, the order of “A” and “D” in a sequence of actions could convey information about examinees’ decisiveness or hesitancy of their decision-making process. For example, the action string “SRAAAAARDE” can be categorized as a meta-strategy “AD sequence,” implying the examinee “draws” the diagrams right after “applying” the simulations on sliders rather than jumping back and forth between applying sliders and drawing diagram lines.

**FIGURE 2:**
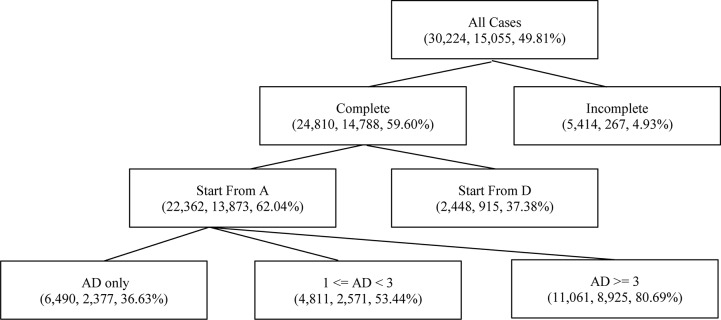
A tree-based diagram for contracted levels of the AD sequence. Indices in parentheses are sample size, number of correct responses, and conditional probability of correctness, respectively, for each class or contracted class of the “AD sequence” variable.

Table 3 shows the distribution of the AD sequence variable, where N indicates the cases in which participants did not conduct an experiment or generate diagrams. Note that the AD sequence’s having an undue number of levels not just hindered interpretation but also caused data sparsity in analysis that followed. Thus a “compact” version of AD sequence with fewer levels was created as shown in Table 4. Figure 2 illustrates how to create the contracted levels in Table 4 by a tree-like diagram.

**TABLE 3:** All levels of AD sequence with sample size and percentage of correctness.

**AD Behavior**	**Total**	**Correct**	**Percentage (%)**
AD	6490	2377	36.63
ADA	1118	522	46.69
ADAD	2996	1648	55.01
ADADA	697	401	57.53
ADADAD	8004	6470	80.83
ADADADA	1648	1459	88.53
ADADADAD	777	558	71.81
ADADADADA	250	188	75.20
ADADADADAD	167	115	68.86
ADADADADADA	64	41	64.06
ADADADADADAD	74	53	71.62
ADADADADADADA	29	17	58.62
ADADADADADADAD	15	8	53.33
ADADADADADADADA	8	6	75.00
ADADADADADADADAD	6	2	33.33
ADADADADADADADADA	7	3	42.86
ADADADADADADADADAD	4	1	25.00
ADADADADADADADADADA	3	1	33.33
ADADADADADADADADADADAD	1	0	0.00
ADADADADADADADADADADADA	1	1	100.00
ADADADADADADADADADADADADA	1	0	0.00
ADADADADADADADADADADADADADA	1	1	100.00
ADADADADADADADADADADADADADAD	1	1	100.00
DA	803	123	15.32
DAD	398	137	34.42
DADA	232	74	31.90
DADAD	190	91	47.89
DADADA	108	40	37.04
DADADAD	345	259	75.07
DADADADA	124	76	61.29
DADADADAD	84	54	64.29
DADADADADA	38	18	47.37
DADADADADAD	22	11	50.00
DADADADADADA	27	7	25.93
DADADADADADAD	11	5	45.45
DADADADADADADA	10	0	0.00
DADADADADADADAD	10	7	70.00
DADADADADADADADA	12	2	16.67
DADADADADADADADAD	6	2	33.33
DADADADADADADADADA	8	0	0.00
DADADADADADADADADADA	3	2	66.67
DADADADADADADADADADAD	1	0	0.00
DADADADADADADADADADADA	3	2	66.67
DADADADADADADADADADADAD	2	1	50.00
DADADADADADADADADADADADA	6	3	50.00
DADADADADADADADADADADADAD	1	0	0.00
DADADADADADADADADADADADADAD	1	1	100.00
DADADADADADADADADADADADADADADA	3	0	0.00
DADADA			
N	5414	267	4.93

**TABLE 4:** All contracted levels of AD sequence with sample size and percentage of correctness.

	**Total**	**Correct**	**Percentage (%)**
Incomplete	5414	267	4.93
Start from D	2448	915	37.38
AD only	6490	2377	36.63
1<=AD<3	4811	2571	53.44
AD>=3	11061	8925	80.69

Process data also provide rich information related to time. Process data includes timestamps of actions, allowing the time spent on a specific action to be calculated by taking the difference of the time of two adjacent actions. Several time-related predictor variables can be generated as follows. “A time” and “D time” indicate the accumulated time spent on manipulating controls and drawing diagrams, respectively. For example, for an action sequence “SADRE,” “A time” is the time used after hitting the “start” box and before hitting the “apply” box; “D time” is the time spent after hitting the “apply” box and before drawing a line among diagrams. By a similar token, “E time” records the time spent after conducting the last action before hitting the “end” box. A special case is “R time,” which represents the time spent after hitting the “reset” box but before conducting the next Action. “time_bf_action” records the time span between “start” and the first action after “start,” which can be considered as the time spent on reading and perceiving the task.

Given the feature generation method described above, 77 variables were created from the process data (a snapshot of the process data is presented as Table 2), as presented in Table 5. Note that time-related features in this study were binned with equal percentiles in terms of their frequencies – the frequency of each bin ranges from 10 to 25% of the sample depending on the variables. This was done essentially due to the nature of the tree models: continuous variables are discretized to find the best “split” point, as discussed in previous sections. This inherent discretization mechanism tends to create data sparsity when the distribution of a continuous variable is “discontinued” (i.e., having extreme low density at the area between modes), which increases the chance of encountering a computation failure. Therefore, to reduce this chance, practitioners “stabilize” the distributions of these “discontinued” variables by binning before feeding the variables to fit the algorithm. In this study, binning was also applied to *n*-gram features with levels having sparse sample sizes. However, it should be noted that binning entails a risk of losing information about these variables.

**TABLE 5:** Variables generated from process data of climate control task 1.

	**Total**	**Generated Features**
Unigram	3	D, R, A
Bigram	16	DD, AA, RA, AR, AD, DA, AE, SD, SA, DR, DE, RD, RE, RR, SR, SE
Trigram	48	ADD, AAR, SRD, DDR, AAE, DRE, AAA, ARD, SDR, ADE, RAA, RRE, DDD, DAR, ARR, DAA, RDA, RRA, DAD, SDA, RRR, AAD, RAD, RRD, ADR, ARE, DRR, RDE, DRR, SRA, ADA, SAR, SRE, ARA, RAR, SDE, DRA, RDD, RDR, SDD, DAE, SAR, DDA, DRD, SRR, SAA, SAD, RAE
Behavioral indicators	4	AD sequence, VOTAT group, VOTAT num, n_actions
Time-related features	6	D time, A time, R time, E time, total time, time_bf_action.
Total	77	

### Feature Selection

Feature selection was conducted using the R package *randomForest* (Liaw and Wiener, 2002). The selection began with seeking the random forest algorithm having the optimal complexity to fit the dataset. In this study, the complexity of the random forest algorithm is characterized by combinations of number of trees (*ntree*) and number of predictor variables used to grow a tree (*mtry*). Empirical studies (Breiman, 2001; Mitchell, 2011; Janitza and Hornung, 2018) showed that *mtry* and *ntree* are more influential than other factors in controlling the complexity of the random forest algorithm. In this study the size of a tree (i.e., the number of generations or the total number of nodes) was not restricted and the number of branches used at each split was fixed at 2. The present study was focused on exploring the combinations of *mtry* and *ntree*, where *ntree* = 100, 300, 500, and *mtry* = 4, 6, 8, 10, 12.

#### Cross-Validation

A typical way to find the optimal model complexity (i.e., the combination of tuning parameters) is to compare the fitted models by their validation error. The validation error is obtained by holding out a subset of the sample (validation set), using the retained sample (training set) to fit the classification algorithm, and then estimating the prediction error by applying the fitted algorithm to the validation set. To efficiently utilize data with a limited size, practitioners (Breiman and Spector, 1992; Kohavi, 1995) have recommended five- or ten-fold cross-validation. In the case of five-fold cross-validation, the data is split into five roughly equal parts. A loop of validations is then conducted – each part is labeled as the validation set once to estimate the prediction error of the random forest model fitted using the other four parts. In a data-rich situation, Hastie et al. (2009) recommended to isolate an additional set (the test set) from the sample before conducting cross-validation. This set is used to compute the prediction error for the final chosen model. It can also be considered as an assessment of the generalization performance of the chosen model on independent data. The present study randomly selected roughly 10% of the sample (3,000 students) as the test set; the rest was separated into five folds for the training-validation purposes.

#### Variable Selection and Backward Elimination

The core idea of validation is to keep the validation sample from being “seen” by the model training process. Such a principle must also be obeyed when variable selection is involved. An example of violating this rule would be to conduct variable selection based on the whole sample before tuning model parameters based on cross-validation (Hastie et al., 2009).

The variable selection implemented in the current study is based on the recursive feature elimination in Guyon et al. (2002) that iteratively rules out features at the lower end of the ranking criterion. Together with random forests, recursive feature elimination has been successfully employed in genome-wide association studies (e.g., Jiang et al., 2009). The variable selection approach suggested in the present study is not just an application of recursive feature elimination using the random forest algorithm with a specific focus on the process data, but a modification with an emphasis on end-to-end cross-validation.

**Box 1. Backward elimination algorithm for feature selection.**

randomly split the training-validation dataset into **5 disjoint subsets**.**X**_1_,…,**X**_5_ are sets of covariates; they are all same with 77 covariates at the beginning;

**repeat** the followings until the covariate sets **X**_1_, …, **X**_5_ are empty:

**for k** in {1,2,…,5}:

hold the **k**-th dataset out for ranking;

**for** each combination of ***mtry*** and ***ntree***:

conduct a five-fold cross-validation using the other 4 datasets and covariates left in **X_k_**;

obtain cross-validated prediction error **e** for the current combination; find the optimal ***mtry*** and ***ntree*** by comparing **e** across all combinations; fit a random forest using the **k**-th dataset and the optimal parameters; obtain the importance rank and remove the least important feature from **X_k_**.

**end**

Box 1 outlines the suggested backward elimination algorithm for variable selection. Note that to prevent variable selection (i.e., ranking) from seeing the data used for model training (i.e., parameter tuning in this study), the training-validation dataset was divided into five disjoint subsets in this recursive selection process so that at each backward elimination parameter tuning can be conducted using four of the subsets of data while variable ranking can be performed separately based on the other subset. This suggested approach follows the principles of variable selection for study design recommended by Brick et al. (2017).

**FIGURE 3:**
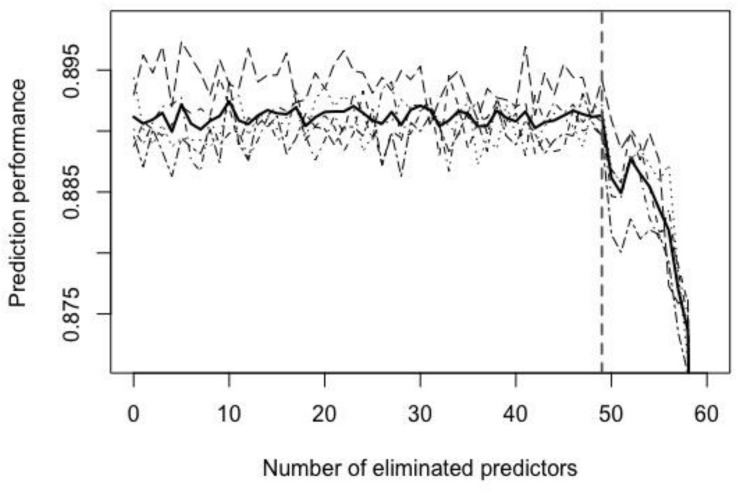
Prediction performance versus number of eliminated predictors for a backward elimination. Dashed lines record the change of validation performance (classification accuracy) for each training set as the number of eliminated feature increases; the bold solid line represents the average performance for five-fold; the vertical dashed line (the number of excluded features=49) indicates where a large reduction of prediction performance begins.

As indicated by Box 1, the backward elimination also documents how the validation performance of the fitted model changes as the number of features reduces, which was illustrated in Figure 3. The number of selected features was decided by drawing a cutoff line around where the first large drop in prediction performance begins (i.e., 49 in Figure 3). Setting this cutoff line here is like selecting the number of factors using the scree plot (Cattell, 1966). Given this threshold number (i.e., 77−49 = 28), five sets with 28 selected features were obtained, and their intersection gives the final selected set of features (26 features).

The backward elimination in Box 1 has five separated iterative variable ranking processes, which could be somehow regarded as an implicit self-validation. However, the determination of the cut-off line shared by the five ranking processes (i.e., the feature screening) should be further validated if data are rich enough. Instead of having one training-validation set, five disjointed training-validation sets (notice this is different from the five shown in Box 1) were established after the test set was held out. Backward elimination shown in Box 1 was conducted for each of the five sets. Accordingly, five sets of final selected features were obtained. Table 6 shows the intersection of these five sets of selected features.

**TABLE 6:** Features selected through the five-fold validated backward elimination.

21 features	D, AD sequence, VOTAT num, **DD**, DDD, VOTAT group, **DDE**, RA, **AD**, R, D time, R time, n_actions, A, AAA, **ADD**, **AR**, **DA**, ADR, **DRA**, **DR**.

*Boldfaced cases indicate features considered redundant. Such features are removed from the set of selected features for analysis that follows.*

The backward elimination in Box 1 was structured using a nested loop that might cause inefficiency. Practitioners can increase the number of features eliminated for each round to reduce computation burden. Plus, as noted by Breiman (2001), the value of *mtry* set around the square root of the number of predictors seems to have minimal effect on validation performance; to increase computational efficiency, one can utilize this deterministic way to adapt the value of *mtry.* In addition, to further increase algorithmic efficiency, researchers (Breiman, 2001; Nicodemus and Malley, 2009; Zhang et al., 2010; Goldstein et al., 2011; Oliveira et al., 2012) recommended employing out-of-bag error as an alternative to cross-validation error. Simulation studies (Mitchell, 2011; Janitza and Hornung, 2018) showed that although out-of-bag error tends to overestimate true error rate when “*n*≪*p*”—that is, the sample size is far less than the number of predictors, the overall validation performance is not substantially affected by means of out-of-bag error to determine model complexity. The present study also performed a backward elimination boosted by using the above suggestions, which obtained consistent results with the plain approach shown in Box 1 in terms of variable selection. Such results were not presented in the manuscript for the sake of simplicity.

## Results

The final set of selected features includes ordinal and binary categorical variables. Pairwise associations among these ordinal variables were measured using the Goodman-Kruskal gamma (γ; Goodman and Kruskal, 1954) with value from −1 (discordant) to 1 (concordant). Given the measure, the final set can be further reduced by removing the redundant features highly related to others.

Among all pairs, “DD” was highly associated with “DDD”(γ = 0.76); “AR” and “RA” was associated with γ = 0.71; other well-associated pairs (γ > 0.6) included “AD sequence” with “AD,” “AD sequence” with “DA,” “AD” with “ADD,” “DRA” with “ADR,” “DRA” with “DR,” and “DD” with “DDE.”^3^ It is not surprising that “AD sequence” was highly correlated with “AD” and “DA.” “AD sequence” was preferred since it covered more information than “AD” and “DA” do, as discussed earlier. “DDD” was greatly associated with “DD;” trigram was preferable in this case since it contained more detailed information. “DDE” conveyed trivial information compared to “DD” and “DDD,” as did “ADD” to “AD.” “AR” and “RA” covered similar information, as did “DRA” with “DR” and “ADR;” the one with higher rank of permutation importance was preferred. In sum, eight features (boldfaced in Table 6) were excluded: “AD,” “DA,” “ADD,” “DDE,” “DRA,” “AR,” “DD,” and “DR.”

With the 13 remaining features, a random forest was fitted with the parameter set where *ntree* = 100 and *mtry* = 4. The parameter combination was chosen based on validation performance of the test set that had been held out at the beginning. Applying the test set here was necessary since the association measured above was based on the entire validation-training sample, which means that variables selected using γ had already “seen” the validation data. Similarly, another random forest was fitted with 77 features; the parameter set was tuned using the test data, where *ntree* = 300 and *mtry* = 9. Here the Goodman-Kruskal tau (τ; Goodman and Kruskal, 1954) was used to measure the proportional reduction of incorrect prediction for the full and the reduced model, respectively, with regard to the random guess based on observed distribution of responses, where τ_77_ = 0.810 and τ_13_ = 0.797. In this regard, the reduced model performed decently in comparison to the full model.

Features of the simple model ranked by the permutation importance measure are shown in Table 7. Unigram “D,” “R,” and “A” ranked high in the list since they are basic elements constituting action sequences. Furthermore, “D” and “R” are not just fundamental but also imply a student’s decisiveness. Using only a few necessary steps of drawing arrows or applying the reset function only a limited number of times might indicate confidence in providing a correct solution. “VOTAT group” and “VOTAT num” are both critical as shown in the list, which is consistent with the results found by Greiff et al. (2015). The top-ranked “AD sequence” indicates that contracting levels shown in Figure 2 work fine in summarizing students’ behaviors on experimenting. Grams such as “AAA,” “ADR,” and “RA” offer interesting perspectives. For instance, students having a large number of “AAA” tended to show certain patterns in their actions: drawing diagrams right after applying experiments (i.e., the level “AD only” in the feature “AD sequence”) and applying the VOTAT scheme across the three sliders. In further investigating these students, we found that they attempted to create an increasing or decreasing slope of the value of temperature or humidity in the display by repeatedly hitting the “apply” box while fixing the sliders at one particular status, indicating a relatively sophisticated behavior of solving the problem. Frequent usage of “ADR” and “RA” indicated participants utilized the reset function to assist their experimenting and exploration on inputs. “D time” and “R time” can be regarded as time spent on deliberation.

**TABLE 7:** Features ranked by permutation importance measure (mean decrease accuracy).

**Feature**	**Mean decrease accuracy**
D	0.199
VOTAT group	0.056
AD sequence	0.042
VOTAT num	0.023
R	0.022
R time	0.018
DDD	0.017
n_actions	0.015
RA	0.014
A	0.013
D time	0.009
AAA	0.008
ADR	0.007

## Discussion

The aim of the present study is to pedagogically suggest an integrated approach to analyze action sequences and other information extracted from process data. Feature generation and selection are two essential parts of the suggested approach and should be treated with equal importance. Features in this study were created following both top-down and bottom-up schemes. The former generates features based on hypotheses that might be developed by item designers and content experts. The latter, as an example, extracts features by utilizing *n*-gram methods and related methods breaking up the action sequences. Thus, *n*-gram translates the action sequences into mini-sequences along with their frequencies. Features generated by both schemes are presented in the final set of selected predictive features. The random forest algorithm was implemented in the feature selection part, which simultaneously handled (1) a massive number of categorical predictor variables, (2) the complexity of the variable structure, and (3) model/variable selection in a computationally efficient way. The utility of the suggested approach has been illustrated by implementing it in a publicly available dataset.

The suggested approach is not free from limitations. First, the feature generation process involves breaking up action sequences into mini-sequences encoded as *n*-grams, suggesting that the information contained in the order of the action sequences – for example, the “longer term” dependencies among actions – would not be completely preserved and exploited. As an outcome, only limited amounts of behavioral indicators are generated; information embedded in students’ action sequences might not be fully utilized. For example, the range of states of controls explored by a student is a variable likely associated with the response variable. Technically speaking, to preserve more “complete” information when analyzing action sequences, sequence-mining approaches (e.g., SPADE; Zaki, 2001) employed to find common subsequences provide a possible alternative. Also, ideas stemming from cognitive and learning studies offer a theoretical basis of creating features from action sequences; for example, some studies (Jiang et al., 2015, 2018) employed sequential pattern mining to analyze learning skills and performance in immersive virtual environments.

Second, most features, if not all, are ordinal categorical variables representing frequency. As noted in the previous section, some variables present in excessive levels could cause an issue of data sparsity when conducting the random forest algorithm. This study used equal-percentile binning to address this issue at the expense of losing information provided by the original variables. The sensitivity of binning needs to be further investigated.

Third, the CART-based random forest algorithm using the Gini-impurity index to split nodes (e.g., the *randomForest* R package used in this study) implemented in this study is generally a suboptimal choice. Strobl et al. (2007) showed that the algorithm tends to favor categorical variables with extensive levels as well as a cluster of variables that are highly correlated. The modified random forest algorithm proposed by Strobl et al. (2007) using the conditional inference tree introduced by Hothorn et al. (2006) should be explored in the context of process data for future studies.

Fourth, even though the efficiency of the suggested backward elimination can be increased by using several steps noted in the previous section, the computation burden is still a concern for the present study. Backward elimination with the specifications shown in Box 1 was validated using a five-fold dataset, which took about 19,872 s in total on a Mac Pro desktop with a 3.5 GHz CPU and 16 GB of RAM.

Fifth, like other data-driven algorithms, the random forest approach is not straightforward regarding model interpretation. For example, hypothesis tests on marginal effects of features are not sustained in random forests; the directions of marginal effects are not directly accessible, either. Friedman (2001) suggested plotting the partial dependence between the feature and the outcome variable (logit is used if the outcome variable is categorical) to access the marginal effects. This display method has been implemented in the R package *randomForest* as the function partialPlot. It is sensible to apply models with more restricted functional forms, such as linear models, to conduct an *ad hoc* analysis based on the selected features.

Sixth, the random forest algorithm is a data-driven method that is sensitive to sample characteristics. Meanwhile, PISA is an international large-scale assessment involving mixed-type forms of tests and multistage sampling designs. The question on how the sampling designs affect the analysis using data-driven methods (i.e., random forests) in terms of estimation stability is beyond the scope of this study. It is appealing that future methodological research could provide guidance concerning the correct use of cross-validation in different test designs.

Last, the exploratory nature of the suggested approach comes with the purpose of the study. Although interesting patterns of behaviors have been found by the suggested approach, it is still difficult to test a cognitive or psychometric theory with it.

The suggested method offers an alternative to the generation and selection of informative features from a massive amount of process data, given the increasing attention to exploring the usage of process data along with response data in large-scale assessments. Generalizability of the method can be explored by applying it to multiple tasks constructed using a similar approach such as MicroDYN and comparing it with other variable-selection approaches in terms of practical significance.

## Ethics Statement

This study is a secondary analysis based on released datasets from PISA 2012 log data files (http://www.oecd.org/pisa/pisaproducts/database-cbapisa2012.htm). No additional data were collected from human subjects for this particular study.

## Author Contributions

ZH contributed to the development of methodology exploration, model estimation procedures, conduction of the data analysis, and drafting and revision of the manuscript. QH contributed to the development of the methodological framework, supervision on the model estimation procedures, conduction of the data analysis, and drafting and revision of the manuscript. MD contributed to providing suggestions on the methodological framework and the model estimation procedures, and reviewing and revision of the manuscript.

## Conflict of Interest

The authors declare that the research was conducted in the absence of any commercial or financial relationships that could be construed as a potential conflict of interest.

## Footnotes

1 Predictor variables and covariates are also used interchangeably without being specifically mentioned in sections that follow.

2 The dataset is available at http://www.oecd.org/pisa/data/pisa2012database-downloadabledata.htm.

3 As a reminder, “D” refers to drawing the diagram, “A” to applying the simulations on the slider, “S” to start, and “R” to reset.
